# Engineered Bacterial Nanosyringes Induce Transient and Controllable Hepatic Immune-Metabolic Responses

**DOI:** 10.4014/jmb.2601.01002

**Published:** 2026-05-25

**Authors:** Xianmei Chen, Yanru Kang, Minghai Shan, Noura M. Bin Yahia, Yanxia Liu, Jiali Yang, Yuma Yang, Mei Hai, Jinyu Li, Feiyan Long, Hao Ma, Shaoqi Yang, Yanhui Yang

**Affiliations:** 1 Department of Gastroenterology, General Hospital of Ningxia Medical University (the First Clinical Medical College of Ningxia Medical University), Yinchuan, Ningxia 750004, P. R. China; 2 Ningxia Key Laboratory of Infection and Immunity, School of Basic Medicine Science, Ningxia Medical University, Yinchuan, Ningxia 750004, P. R. China; 3 Department of Gastroenterology, People's Hospital of Ningxia Hui Autonomous Region, Ningxia Medical University (the Third Clinical Medical College of Ningxia Medical University), Yinchuan, Ningxia 750004, P. R. China; 4 Ningxia Medical University, Yinchuan, Ningxia 750004, P. R. China

**Keywords:** Photorhabdus virulence cassette, Contractile injection systems, Liver, Transcriptomics, Inflammation

## Abstract

The photorhabdus virulence cassette (PVC), a programmable protein-delivery nanodevice derived from bacterial contractile injection systems, can be engineered to possess recognizing and targeting human cells. However, whether it impacts mammalian liver function and transcriptional networks remains largely unknown. Engineered PVCs were injected intraperitoneally into BALB/c mice. Hepatic transcriptomes were analyzed by RNA sequencing (RNA-seq) and Gene Ontology/Kyoto Encyclopedia of Genes and Genomes (GO/KEGG) clustering was applied to differentially expressed genes (DEGs) in RNA-seq analysis of the liver's transcriptomes. No acute hepatotoxicity was seen, as indicated by unchanged plasma alanine transaminase (ALT) and aspartate aminotransferase (AST). RNA-seq identified 6,471 DEGs compared with the 0 h group, early transcriptional responses in (2-4 h) group were dominated by innate immune pathways (NF-κB, TNF, and chemokine), driven by upregulation of pattern-recognition receptors (*e.g.* *Tlr2*, *Myd88*, and *Cd14*) and cytokines. In 12-24 h group, analysis of the significantly downregulated genes showed that multiple genes, such as CYP450, glutathione S-transferases, and carboxylesterase family genes were markedly decreased at 12 h, most of the genes gradually approached 0 h. Time-series analysis revealed coordinated immune-metabolic dynamics: acute inflammation (bell-shaped trend) followed by metabolic reprogramming (U-shaped trend). Single intraperitoneal injection of PVC induces a transient and controllable immune-metabolic response without obvious hepatotoxicity.

## Introduction

Contractile injection systems (CIS) are new macromolecule machines that target effector proteins to cells using a conserved contractile sheath-tube mechanism[1]. The mechanism of CIS function is spring-like contraction of the outer sheath, which drives the inner tube through target cell membranes and directs delivery of encapsulated cargo molecules directly from the cell membranes[2, 3]. Phage-derived CISs such as T4 tail and R-type pyocins have been characterized both structurally and functionally[4, 5]. However, recent focus has been on extracellular CISs (eCISs) from bacteria, particularly the PVC, which has several advantages for biomedical engineering[6].

The PVC system represents a particularly promising eCIS variant due to its relatively simplified architecture, expansive luminal capacity accommodating diverse cargo types, and innate capacity for receptor-specific targeting through genetically encoded tail fiber modifications. These inherent properties have facilitated the engineering of PVC into a programmable protein delivery platform capable of transporting functional effectors to specific cell types [7]. In 2025, the notable development of the SPEAR (Spike Engineering And Retargeting) system has substantially expanded the PVC platform's utility through rational design of spike proteins that enable precise retargeting to designated cell surface receptors while accommodating an increasingly diverse repertoire of cargo molecules, including base editors and single-stranded DNA[8]. This engineering flexibility positions PVC-based systems as attractive alternatives to conventional delivery modalities for therapeutic applications.

The liver is a particularly important evaluation site for safety evaluation of systemically administered delivery systems because it plays a key role in xenobiotic metabolism, blood clearance, and immune response [9, 10]. The liver is the main filter of circulating particulates and macromolecules, and the hepatic response can play a crucial role in the translational feasibility of xenobiotics[11]. PVC-based systems have shown promising targeting efficiency and cargo transfer capabilities in preliminary studies[7], but their impact on hepatic function and global transcription networks is still not well understood. Even small perturbations of liver enzyme activity or induction of inflammation may also play an important role in clinical translation [12]. In addition, understanding how eCISs modulate time-dependent gene expression patterns in the liver could reveal fundamental host response mechanisms to this novel class of PVC for safety assessment and future engineering optimization.

We characterized the acute response of the liver to PVC in a murine model by analyzing serum biomarkers, inflammation factors, and temporal transcriptomic profiles. We show that intraperitoneal injection of PVC induces rapid but self-resolving transcriptional reprogramming that encompasses both the upregulation of immune genes and the downregulation of metabolic genes. In addition, it was a transient response with no hepatocyte damage with ALT/AST stability and resolution within 24 h. These results indicate that PVC triggers a transient and controllable hepatic immune-metabolic response. These findings provide experimental support for the use of PVC as a vector in mammalian systems.

## Materials and Methods

### Plasmid Expression and Purification

The PVC structural and accessory region (pAWP78-pvc13-Ad5Knob, 198289) and payload and regulatory region (pBR322-Pdp1-NTD-Cas9, 198275) were purchased from the Addgene company. Both of the plasmids were transfected into EPI300 cells. Colonies were inoculated into 2 mL of Terrific Broth and cultured with shaking at 37°C for 16 h. The cultures were transferred to 500 mL of Terrific Broth medium at a 1:1,000 inoculation ratio, followed by a further 24 h of shaking incubation at 37°C. After the incubation period, the cultures were centrifuged at 4,000 × g for 30 min, and the resulting pellets were resuspended in 28 mL of lysis buffer (25 mM Tris-HCl pH 7.5, 5 mM MgCl_2_, 3 mM KCl, 140 mM NaCl, 50 μg·mL^-1^ DNase I, protease inhibitor cocktail, and 0.5% Triton X-100). Subsequently, the mixture was shaken at 37°C for 90 min to facilitate cell lysis. After lysis, we carried out the purification of PVC by means of ultracentrifugation. The purification procedure settings were consistent with prior descriptions [7]. Finally, the sample was resuspended in 15 mL PBS, followed by concentration with an Amicon^®^ Ultra 100 kDa device (4,000 × g, 30 min) to remove small-molecule substances. The amount of PVC produced was determined via A280 measurement using a NanoDrop. The endotoxin levels were then quantified using a Pierce Chromogenic Endotoxin Quant Kit (Beyotime Biotechnology, China) and determined to be below 0.1 EU/mg.

### Electron Microscopy

Transmission electron microscopy (TEM) analysis of purified PVC particles was carried out at Ningxia Medical University's Electron Microscopy Laboratory. A PVC sample was added in 5-10 μL onto a 200-mesh carbon-coated copper TEM grid and incubated for 5 min. Excess liquid was blotted with filter paper, followed by staining with 2% phosphotungstic acid for 30 sec. The grids were air-dried at room temperature before imaging using a Hitachi Limited H-7650 transmission electron microscope.

### Mouse Infection and Sample Collection

The experimental mice were 12-week-old specific pathogen-free female BALB/c mice, weighing 18-22 g. The mice were housed in a standard environment (temperature of 22-26°C and humidity of 40%-60%), and the light cycle was controlled as 12 h of light (from 8:00 to 20:00). All animal experiments were strictly conducted in accordance with the guide for the care and use of laboratory animals and approved by the Animal Experimental Ethics Committee of Ningxia Medical University (No: IACUC-H2025127).

Mice were subjected to a 1-week acclimatization period before the experiment. Each mouse received an intraperitoneal injection of 300 μL untargeted PVC (0.20 μg/μL) in sterile 0.9% NaCl. The mice were deeply anesthetized and then sacrificed by cervical dislocation at 0 h, 2 h, 4 h, 12 h, and 24 h after administration, respectively. The liver tissue and plasma were collected, immediately stored, and kept at -80°C until further processing and analysis.

### Library Preparation and Sequencing

Total RNAs were extracted from tissues using TRIzol^®^ Reagent according to manufacturer's instructions. RNA concentration was measured with ND-2000 spectrophotometer (NanoDrop Technologies, USA) and RNA integrity was measured with 5300 Bioanalyzer (Agilent, USA). Only RNA samples passed quality control thresholds (OD_260/280_ = 1.8-2.2, OD_260/230_ ≥ 2.0, RQN ≥ 6.5, the ratio of 28S:18S ≥ 1.0) were used for library construction.

RNA purification, cDNA synthesis, library construction, and sequencing were carried out by Shanghai Majorbio, China. The mice’s liver RNA-seq transcriptome library was prepared using the Illumina Stranded mRNA Prep Ligation Kit with 1 μg total RNA input. Polyadenylated mRNA was isolated, fragmented, and used for double-stranded cDNA synthesis. End repair, phosphorylation, and adapter ligases were performed according to the manufacturer’s instructions. Libraries were size-selected for 300 bp fragments, and 15 cycles of polymerase chain reaction (PCR) were performed. Quantification was performed with a Qubit 4.0 fluorometer, followed by paired-end sequencing (PE150) on the NovaSeq X Plus platform with the NovaSeq Reagent Kit, USA.

### Quality Control and Read Mapping

Transcriptome analysis on 15 samples yielded 97.94 Gb of clean data. Clean data volume reached more than 5.9 Gb, and Q30 base was more than 96.1% (Table S1). We aligned clean reads with the reference genome (GRCm39, http://asia.ensembl.org/Mus_musculus/Info/Index) orientation-aware using HISAT2. StringTie was used to construct the reference-based reads of each sample.

### Analysis of Differential Expression and Functional Enrichment

We employed the transcripts per million reads method to quantify transcript expression levels and identify DEGs between sample pairs, with gene-level abundance estimation performed by RSEM (http://deweylab.github.io/RSEM)[13]. The analysis of differential expressions were carried out using either the DESeq2 method. Genes with a log2FC greater than 1.0 or less than -1.0, and an FDR (False Discovery Rate) less than 0.05 were considered significantly differentially expressed. GO and KEGG pathway analysis were employed for functional enrichment and pathway analysis.

### Histopathological Analysis

All tissues were fixed in 10% formalin solution, dehydrated in a graded ethanol series, and embedded in paraffin. Serial sections of 2 μm thickness were prepared and stained with hematoxylin and eosin (H&E).

### ALT, AST, and Inflammatory Cytokine Measurements

For the purpose of analyzing the biochemical index, the plasma was separated from the whole blood using a chilled centrifuge. The ALT and AST activities of the serum were estimated by using commercial kits (China). Liver tissue homogenates were prepared and measured for IL-6, TNF-α and IL-1β by commercial ELISA kits according to the manufacturer's instructions (Proteintech, China).

### Validation of RNA-Seq Data

The liver tissue was used to extract total RNA. Using the primer sequences listed in Table 1, a subset of six genes was chosen for quantitative real-time reverse transcription PCR (qRT-PCR) validation from the RNA-Seq statistical analysis. The 2*^−ΔΔCT^* method was used to calculate the gene expression levels, and the findings were shown as log2 fold changes.

### Western Blot

Total protein from liver tissues was extracted by RIPA, and western blotting analysis was performed as previously described [14]. The antibodies for Anti-MTHFD1 (EPR29810-508), Anti-ICAM1 (EPR16608), MyD88 (4283), β-Actin was used as invariant controls for equal loading of total proteins. The bolt were quantified using ImageJ software (version 1.41).

### Statistical Analysis

The mean ± standard error of the mean (SEM), which was obtained from several samples or at least three technical replicates, was used to express all data. Two-way ANOVA was used for datasets with multiple measurements per mouse. FDR, method was used to adjust for multiple comparisons in this analysis, with a predetermined *P*-value of less than 0.05 was deemed statistically significant in all statistical analyses. The statistical analysis mentioned above was performed using SPSS software (version 22.0) and GraphPad Prism (version 9.0).

## Results

### Observation of PVC by Electron Microscopy

**Fig. 1:**
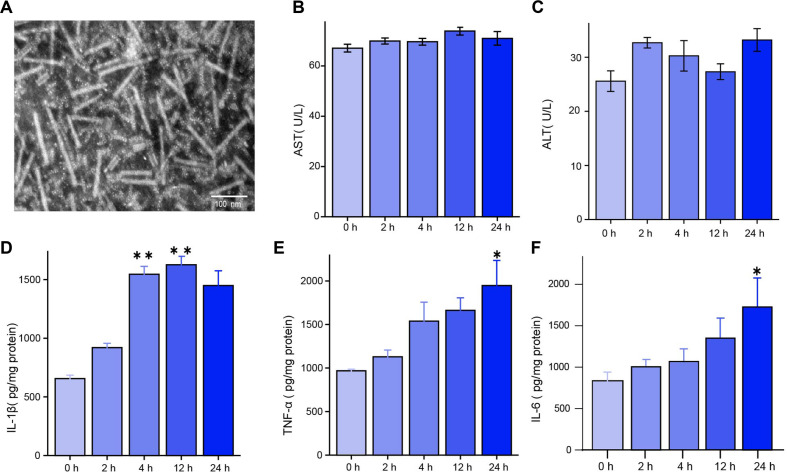
Morphological characteristics of PVC and its effects on ALT, AST, and inflammation. (**A**) The morphology of the purified PVC was observed using the negative-stain TEM. (**B, C**) Serum levels of ALT and AST, *p* > 0.05 vs. 0 h. (**D, E, F**) Liver levels of TNF-α, IL-1β, and IL-6 were determined by using ELISA. Statistical significance among biologically independent samples was calculated via two-way ANOVA followed by Benjamini-Hochberg false method to adjust for multiple comparisons. The data were presented as means ± SEM. Significant differences between groups were indicated as *p < 0.05 vs. 0 h, **p < 0.01 vs. 0 h.

We used double-staining electron microscopy to observe the structural integrity of PVC, and the obtained proteins exhibited intact baseplates and sheath structures with a length ranging from 90 nm to 160 nm (Fig. 1A).

### The Effects of PVC on ALT, AST, and Inflammatory Cytokines

The ALT and AST activities in the plasma are commonly used to assess liver injury. As shown in Fig. 1B and 1C, plasma ALT and AST activities in different time points (2 h, 4 h, 12 h, 24 h) were not significantly different by comparison with those in the 0 h group (*P* > 0.05), suggesting liver function was not significantly damaged.

It has been well-acknowledged that inflammation plays an essential role in PVC infection. Compared with the 0 h group, inflammatory cytokines such as TNF-α, IL-1β, and IL-6 are significantly elevated in hepatic tissue, as measured by ELISA (IL-1β levels significantly increased at 4 h and 12 h, while TNF-α and IL-6 levels significantly increased at 24 h (*P* > 0.05) (Fig. 1D, E, F).

### Time-Dependent Hepatic DEG Profiles Induced by PVC

**Fig. 2:**
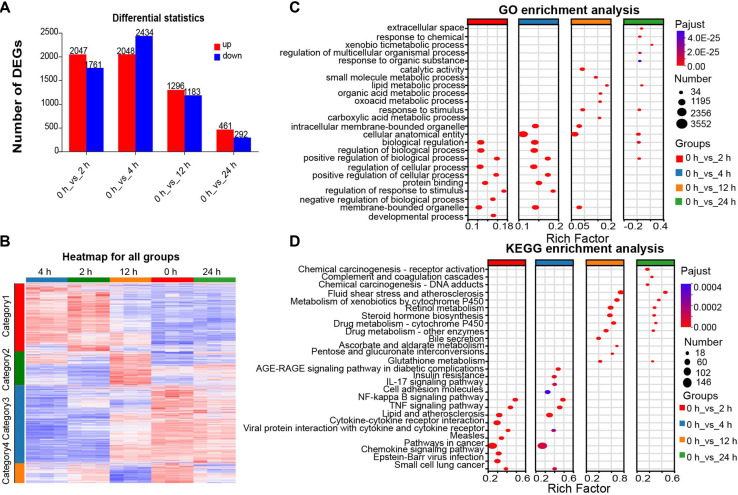
Analysis of differences between the baseline group (0 h) and those at different time points after intraperitoneal injection of PVC. (**A**) The number of DEG that were upregulated or downregulated at different time points compared to 0 h group, The red color represents gene upregulation, and the blue color shows gene downregulation (log_2_FC|≥1) and *p* < 0.05). (**B**) The genes with differential expression at each time point are displayed in a heatmap. The relatively high and low are represented by the red and blue colors. Different categories are represented by the different colors on the left. The categories are as follows: red is the first category1, followed by green, blue, and yellow. (**C, D**) DEGs were subjected to KEGG and GO enrichment analysis following intraperitoneal PVC injection. The number of genes in this pathway is clearly indicated by the dot size, and significant p-values are indicated by the red intensity.

To investigate the transcriptomic characteristics between the 0 h group and the groups at different time points (2 h, 4 h, 12 h, and 24 h) after PVC injection, RNA-seq was performed on liver tissue from 15 mice (n = 3 per group) at the aforementioned 5 time points. The online software DESeq2 and RSEM were used for differential gene expression analysis, with the criteria of DEGs with |log_2_FC| ≥1 and FDR< 0.05 and *p* < 0.05. A total of 6,471 DEGs had an absolute change (Fig. 4A) compared with the 0 h. A further comparison and analysis of the differences between the 0 h group and the 2 h group showed there were 2,047 upregulated DEGs and 1,761 downregulated DEGs; 2,048 DEGs were upregulated and 2,434 were downregulated in 4 h; 1,296 DEGs were upregulated and 1,183 were downregulated in 12 h; the number of DEGs decreased significantly, with 461 upregulated DEGs and 292 downregulated DEGs in 24 h (Fig. 2A).

### Cluster Analysis and Functional Enrichment of Hepatic DEGs Post-PVC Injection

All DEGs generated by comparing each time point with the 0 h group were clustered (Fig. 2B). Based on the upward or downward change trends, they were collectively clustered into 4 categories. Category 1: Significantly increased at 2 h and 4 h, followed by a downward trend at 12 h, and gradually returned to the baseline level at 24 h. Category 2: Similar to the baseline level at 2 h and 4 h, increased at 12 h, and then gradually decreased at 24 h, approaching the baseline level. Category 3: Showed a downward trend at 2 h and 4 h, increased at 12 h, and approached the baseline level. At 24 h, Category 4: Close to the baseline level at 2 h, gradually decreased at 4 h and 12 h, and gradually approached the baseline level at 24 h.

Through GO and KEGG enrichment analyses (Fig. 2C and 2D): Early stage (2 h, 4 h) GO enrichment: Mainly concentrated in functions such as “biological regulation” and “regulation of cellular process”. Early stage KEGG enrichment: Signaling pathways, including NF-κB, TNF, and Chemokine pathways, were significantly enriched. These pathways are mainly involved in inflammatory and innate immune responses during exogenous virus invasion [15, 16].

In 12 h and 24 h, compared with the 0 h group: GO enrichment: Mainly focused on metabolic pathways such as “lipid metabolic process” and “xenobiotic metabolic process”. KEGG enrichment: Metabolic-related pathways, including steroid biosynthesis, metabolism of xenobiotics by cytochrome P450, and Retinol metabolism underwent significant changes. Metabolic reprogramming is a key participant in the immune response to viral infection[17, 18].

### Analysis of DEGs at Different Time Points Compare with Baseline Group (0 h) for Liver Tissue

**Fig. 3:**
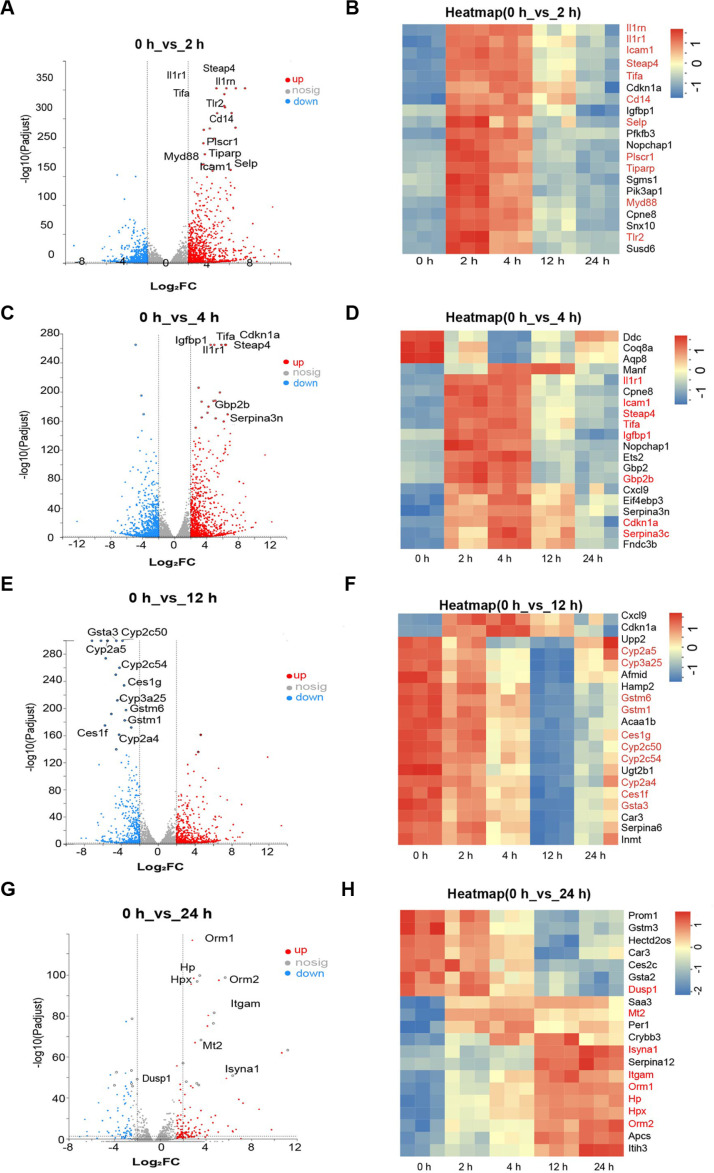
Differential gene expression between 0 h and several time intervals after PVC exposure. (**A**) 0 h vs 2 h, (**C**) 0 h vs 4 h, (**E**) 0 h vs 12 h, and (**G**) 0 h vs 2 h, is plotted as a volcano. Plotting of genes is done using |log_2_FC| ≥ 2. Genes that are upregulated are indicated by red dots, downregulated by blue dots, and non-significant by gray dots, The top 20 up-/downregulated genes in the figure, the key genes have been annotated in text. (**B, D, F, H**) Heatmap showing significant gene expression patterns at each time point after PVC exposure. The color scale depicts relative expression levels, with red being high expression and blue denoting low expression, Key genes were highlighted in red font for emphasis.

To understand which genes changed at each time point and their potential impacts, the top 20 DEGs with significant changes (based on P-adjust) in each group compared to the baseline group were analyzed (Fig. 3A-3F and D, Table 2, and Table S2).

At 2 h, among the genes with persistently high expression, *Steap4* (STEAP family member 4), *IL1rn* (interleukin 1 receptor antagonist), *IL1r1* (interleukin 1 receptor 1), Tifa (TRAF-interacting protein with forkhead-associated domain), *Tlr2* (toll-like receptor 2), *Cd14* (*Cd14* antigen), *Plscr1* (phospholipid scramblase 1), *Myd88* (myeloid differentiation primary response gene 88), *Tiparp* (TCDD-inducible poly (ADP-ribose) polymerase), *Selp* (selectin, platelet), and *Icam1* (intercellular adhesion molecule 1) are closely associated with inflammation and innate immunity (Fig. 3A). Notably, *Tlr2*, *Cd14*, and *Myd88* are critical genes in the NF-κB signaling pathway that mediates inflammatory responses, The clustered heatmap indicates that these genes maintained high expression at 2 h and 4 h, then decreased at 12 h and 24 h, gradually approaching the baseline level (Fig. 3B). At 4 h, some genes that were upregulated at 2 h continued to be highly expressed (Fig. 3C and 3D). Moreover, among the newly significantly upregulated genes at 4 h (*Cdkn1a*, *Gbp2b*, and *Serpina3n*), all are involved in innate immune and inflammation processes (Fig. 3C and 3D).

After 12 h, downregulated genes became predominant. The expression of multiple CYP450 genes (*Cyp2a4*, *Cyp2a5*, *Cyp2c50*, *Cyp2c54*, and *Cyp3a25*) was significantly reduced; genes of the glutathione S-transferase (GST) family (*Gsta3*, *Gstm1*, and *Gstm6*) and genes of the carboxylesterase (*CES*) family (Ces1f and Ces1g) showed decreased expression (Fig. 3E and 3F). After 24 h, this genes gradually approaching the baseline level (0 h). However, differential expressions were still observed in some genes: those involved in inhibiting inflammatory responses (*Orm1* (orosomucoid 1) and *Orm2* (orosomucoid 1)) and those related to maintaining cellular metabolism (Isyna1 (myo-inositol 1-phosphate synthase A1)), *Mt2* (metallothionein 2), and so on (Fig. 3G and 3H).

### Analysis of DEGs Related to Sustained Changes within 24 h

**Fig. 4:**
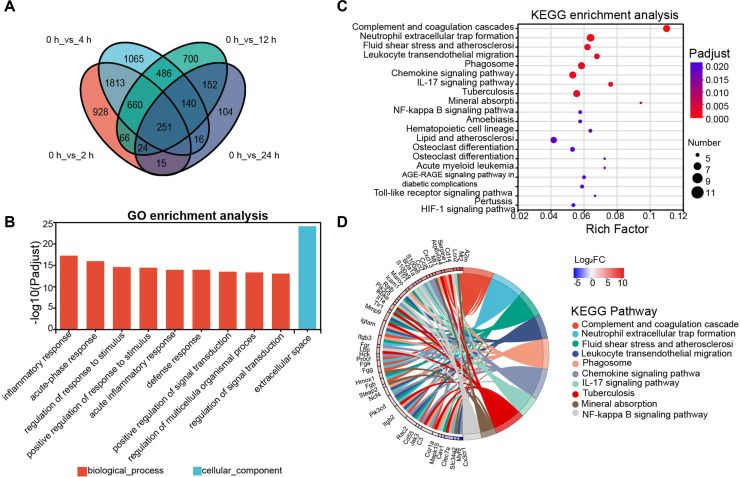
DEGs related to sustained changes within 24 h. (**A**)The numbers on the Venn diagram represented unique and common DEGs in four groups compared to the baseline group. (**B, C**)Functional enrichment analysis of common DEGs (251 genes) in four groups was performed in mouse liver compared to the baseline group (0 h) using GO and KEGG. We identified 10 significantly enriched pathways (FDR < 0.05) based on all significantly differential genes (*P*-adjust < 0.05 and |Log_2_FC| ≥ 1). (**D**) The top ten genes that are significantly associated with the top ten KEGG pathways.

A Venn diagram (Fig. 4A) was used to identify the genes that showed differential expression and sustained changes within 24 h compared to the baseline group. There were 251 genes with statistical differences across all four groups. According to the GO enrichment analysis, these DEGs were primarily enriched in terms of extracellular space, inflammatory response, acute-phase response, and stimulus regulation (Fig. 4B). The KEGG enrichment analysis revealed significant enrichment of pathways including Complement and coagulation cascades, Neutrophil extracellular trap formation, Fluid shear stress and atherosclerosis, and others (Fig. 4C). Meanwhile, and We also examine each KEGG pathway's top ten genes (Fig. 4D). These findings suggest that after entering the liver, PVC primarily stimulates the body's innate immune defense and inflammatory responses.

### Time-Course Gene Set Analysis

**Fig. 5:**
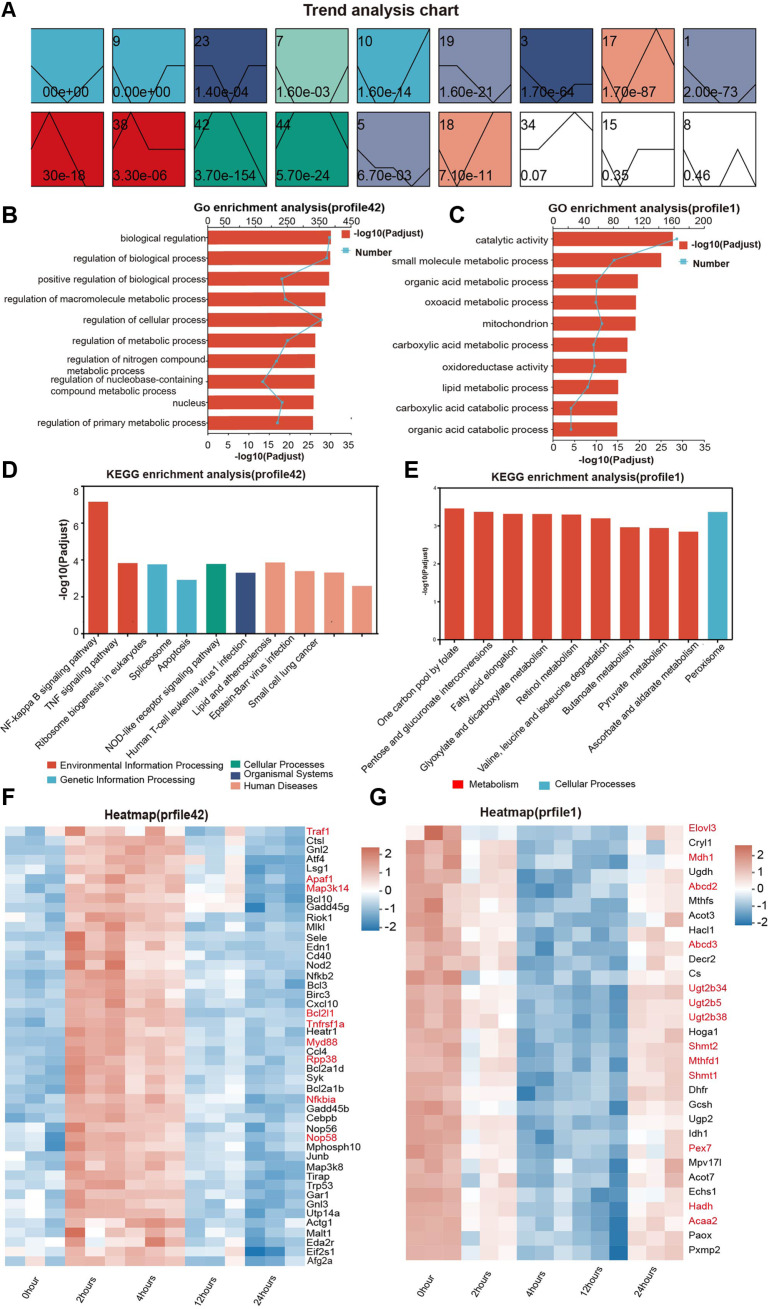
Time-course gene set analysis. (**A**) Schematic of gene expression trend profiles generated by the STEM algorithm.Profile ID: A numerical label located at the top-left corner of each panel.Expression Trend Line: A line within each panel that illustrates the dynamic change of gene expression levels over the time course.Statistical Significance: The *P*-value, displayed at the bottom-left corner of each panel, indicating the significance level of the corresponding trend. (**B, C**) This plot illustrates the results of GO enrichment analysis for genes in profile 42and profile1. (**D, E**) This bar plot illustrates the results of KEGG pathway enrichment analysis for genes in profile 42 and profile1. (**F, G**) Heatmap of Target Gene Clustering Analysis (Profile42 and Profile1), This heatmap illustrated the clustering analysis of target genes (listed on the y-axis) across different samples (labeled on the x-axis). The color scale represented the relative expression levels of genes, with red indicating higher expression and blue indicating lower expression. Hierarchical clustering was performed to identify patterns of gene expression similarity among samples and genes.

To identify genes exhibiting consistent longitudinal changes, we employed the Short Time-series Expression Miner (STEM) algorithm to generate trend plots of genes with altered expression patterns (Fig. 5A). Based on *P*-values and the trends observed in the current experimental results, profile 42 (*p* = 3.7 × 10^-154^) and profile 1 (*p* = 2.0 × 10^-73^) were selected for further analysis. Specifically, Profile 42, characterized by an initial upregulation followed by a rapid decline, was consistent with our previous observation that inflammation-associated genes exhibited an upregulation-then-downregulation pattern. While Profile 1, which displayed a delayed downregulation, mirrored the adaptive metabolic reprogramming observed in the liver following exposure.

Profile 42 displayed a bell-shaped curve characterized by an “initial increase followed by a decrease (Fig. 5A)”. Go enrichment analysis revealed significant enrichment in biological regulation, regulation of biological process, and positive regulation of biological process (Fig. 5B). KEGG enrichment analysis revealed that four pathways—the NF-κB signaling pathway, Tumor Necrosis Factor Signaling (TNF) signaling pathway, apoptosis, and ribosome biogenesis in eukaryotes—were significantly co-enriched (Fig. 5D). From the heatmap, we can see that genes related to the NF-κB signaling pathway (including *Myd88*, *Nfkbia*, and *Map3k14*) were rapidly activated within 2 h to 4 h (Fig. 5F). After that, the genes gradually recover after 24 h (Fig. 5F). The high expression of *Tnfrsf1a* (tumor necrosis factor receptor superfamily, member 1a) and *Traf1* (TRAF-interacting protein with forkhead-associated domain) stimulation of the NF-κB and other downstream signaling pathways amplified the inflammatory response, while their expression showed a downward trend after 12 h. From 2 h to 4 h, *Bcl2l1* (an anti-apoptotic gene) and *Apaf1* (a pro-apoptotic gene) were upregulated synchronously. In the “Ribosome biogenesis in eukaryotes” pathway, key genes including Nop58 (NOP58 ribonucleoprotein) and Rpp38 (ribonuclease P/MRP 38 subunit) exhibited a pattern of initial upregulation followed by downregulation (Fig. 5F).

In the analysis of Profile 1, the expression of liver metabolic genes exhibited a U-shaped curve characterized by “sustained downregulation from 2 h to 12 h → partial recovery at 24 h (Fig. 5A)”. From 2 h to 12 h: Key pathways were significantly suppressed, including the one-carbon pool by the folate pathway (genes: *Mthfd1* and *Shmt1/2*), the pentose & glucuronate interconversions pathway (genes: *Ugt2b34/5/38*), the peroxisome pathway (genes: *Abcd2/3* and *Pex7*), the fatty acid elongation pathway (genes: *Elovl3* and *Acaa2*), and the glyoxylate & dicarboxylate metabolism pathway (genes: *Hadh* and *Mdh1*) (Fig. 5E and 5G). GO enrichment also showed significant enrichment of metabolic pathways (Fig. 5C). The heatmap (Fig. 5G) showed an overall blue color, indicating downregulated gene expression. The colures of most genes shifted from blue to orange in the heatmap, and this indicated that the genes’ expression began to recover at 24 h. However, the expression levels did not fully return to the baseline. This indicated that metabolic reprogramming had entered the “repair initiation” phase, while the liver metabolic function remained lower than the normal level.

### Histopathological Evaluation of Major Organs

**Fig. 6:**
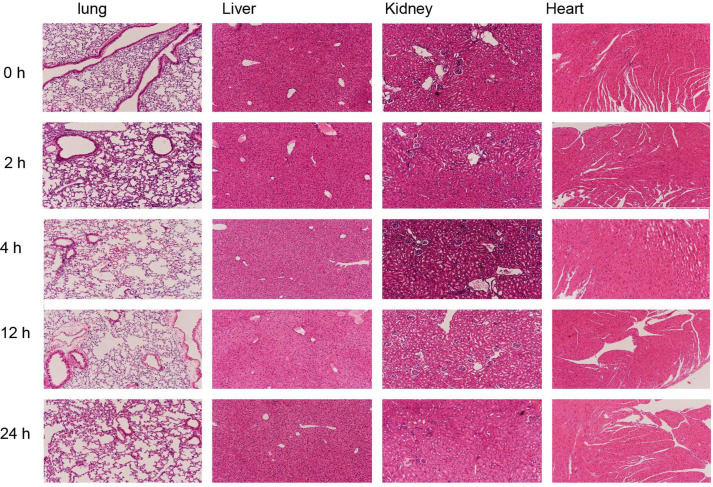
Representative hematoxylin and eosin (H&E) staining images of major organs (lung, liver, kidney, heart) at different time points (0 h, 2 h, 4 h, 12 h, 24 h, 72 h) after intraperitoneal injection of PVC (magnification, 100X).

HE staining showed that the lung tissue structure was intact and well-organized, with uniformly sized alveoli and thin alveolar walls without thickening or disruption. The capillary network in the alveolar septa was abundant, with no congestion, hemorrhage, or inflammatory cell infiltration. No edema, fibrosis, or inflammatory lesions were observed in the pulmonary interstitium.In normal liver tissue stained with hematoxylin and eosin (HE), the hepatic lobular structure was intact, with the central vein located in the center of the lobule. Hepatocytes were radially arranged with regular morphology, centrally located nuclei, and eosinophilic cytoplasm. The portal areas were clearly defined, with no inflammatory cell infiltration or fibrosis observed. The renal capsule was intact, and the boundary between the renal cortex and medulla was distinct. Glomeruli exhibited normal structure, and renal tubular epithelial cells showed normal morphology without vacuolar degeneration, necrosis, or cast formation. No edema, hemorrhage, or inflammatory cell infiltration was present in the renal interstitium, and no obvious vascular lesions were detected (Fig. 6).

### qRT-PCR Validation of RNA-Seq Data

**Fig. 7:**
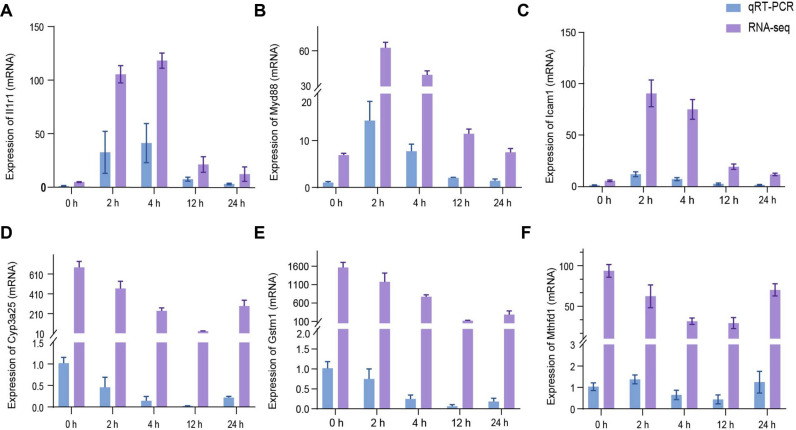
qRT-PCR was used to confirm the relative expression level. From the transcriptome analysis results, the differentially expressed genes about metabolism and inflammation were verified, and their expression levels were assessed. GAPDH expression was used as the reference to normalize the expression levels, The CT value variation of GAPDH is less than 5%. Purple indicates the RNA-seq results, and blue indicates the qRT-PCR results. The CT value variation of GAPDH is less than 5%. The data were presented as means ± SEM (n = 3).

To validate the results of transcriptome sequencing, a total of six genes were selected for qRT-PCR analysis. To verify the effects of PVC on liver inflammation and metabolism in mice, relevant genes that played key roles in inflammatory pathways and metabolism were chosen for validation. In addition, the consistency between qRT-PCR results and RNA-seq results was compared. As shown in Fig. 6A-6C the expression levels of *Il1r1*, *Myd88*, and *Icam1* that the key genes in inflammatory pathways were upregulated in the 2 h and 4 h infected samples but downregulated in the 12 h and 24 h ones. Fig. 7D-7F showed the changing trends of *Cyp3a25*, *Gstm1*, and *Mthfd1* at different time points, which is relative metabolism in liver tissue. The qRT-PCR validation revealed that the expression levels of the selected genes were consistent with those from RNA-Seq.

### Quantification of Protein Expression by Western blot

**Fig. 8:**
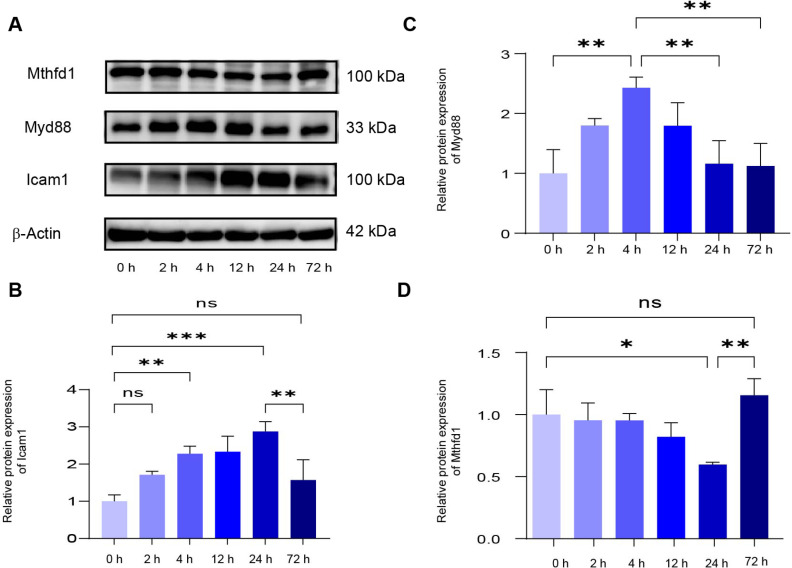
Western blotting used for quantification of Myd88, Icam1 and Mthfd1.

To verify whether alterations at the transcriptomic level resulted in corresponding changes at the protein level, we selected the inflammation-related proteins MyD88 and *Icam1*, as well as the metabolism-related protein Mthfd1, for validation by Western blot. The results showed that the expressions of MyD88 and *Icam1* exhibited a trend of increasing first and then decreasing over time, whereas Mthfd1 expression decreased and gradually returned to baseline levels (Fig. 8).

### Transcriptomic Sequencing Analysis at 72 h Post-Injection of PVC

**Fig. 9:**
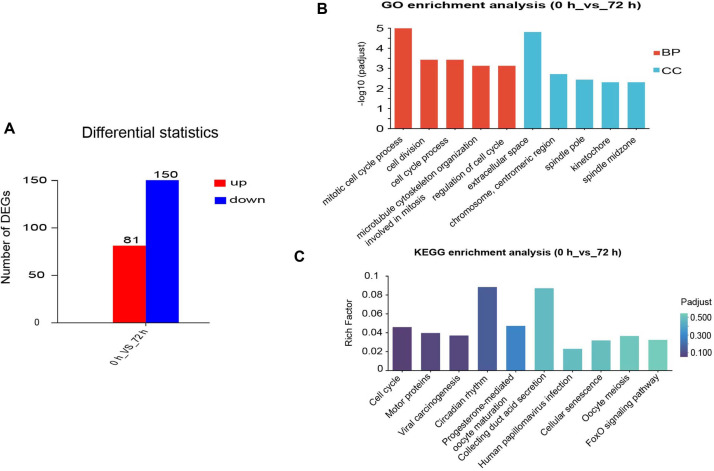
Transcriptomic analysis of differential gene expression and functional enrichment between 0 h and 72 h. (**A**) Bar chart showing the number of DEG that were upregulated or downregulated in the 0 h vs 72 h comparison. (**B**) GO enrichment analysis of DEGs, with top enriched biological processes (BP, red) and cellular components (CC, blue). (**C**) KEGG pathway enrichment analysis of DEGs, with the color gradient indicating the adjusted P-value (Padjust) and bar height representing the rich factor.

Given that 753 differentially expressed genes (DEGs) remained dysregulated at 24 h, we performed additional transcriptomic sequencing at 72 h post-injection to further characterize the sustained transcriptional response. Compared with the 0 h control group, a total of 231 DEGs were identified in the 72 h treatment group, consisting of 81 significantly upregulated genes and 150 significantly downregulated genes. GO enrichment analysis revealed that these DEGs were primarily enriched in biological processes related to the mitotic cell cycle and cell division, as well as in cellular components such as the spindle and nucleolus (Fig. 9). KEGG pathway analysis further confirmed significant enrichment in the cell cycle, motor pioteins,and viral carcinogenesis. Notably, these transcriptional changes were unrelated to inflammation or metabolic processes.

## Discussion

As a novel protein delivery system, PVC exhibits advantages such as high efficiency, programmability, and strong targeting ability. However, most relevant studies are limited to cellular experiments, and PVC’s effects on the mammalian liver have not yet been reported. Here we show the acute hepatic response to PVC administration, demonstrating its transient transcriptional impact without liver injury. Through systematic evaluation of a murine model combining serum biomarkers and temporal transcriptomics, the results show a biphasic immune-metabolic reprogramming that mostly resolves within 24 h. These results provide experimental data for the development of PVC as a programmed protein delivery platform.

The results show that the liver responds to PVC in a biphasic manner: early immune upregulation followed by transient metabolic downregulation. Within 2-4 h of exposure, we detected rapid upregulation of the innate immune components (*Tlr2* and *Cd14*)[19], pattern recognition receptors (*Myd88* and *Tifa*)[20], effector molecules (cytokines (*Il1r1* and *Il1rn*) [21], and adhesion molecules (*Icam1* and *Selp*), and pathway analysis revealed enrichment of TNF, and NF-κB signaling cascades[20], and such alterations frequently occur during the host recognition of viruses or bacteria[19, 22–24]. The concomitant up-regulation of *Il1rn*—an endogenous competitive antagonist of IL-1β,—demonstrates that an autoregulatory loop is already engaged at the initiation step, preventing unrestrained IL-1 signaling[21]. Other genes that are upregulated within 2 h, such as *Steap4* and *Plscr1*, still require further research data to confirm their functions.

In the present study, transcriptomic sequencing data showed that inflammation-related mRNAs started to increase and reached peak levels at 2 h and 4 h, then gradually declined, with most returning to baseline by 24 h. However, the peak elevations of the measured inflammatory cytokines TNF-α, IL-1β, and IL-6 were significantly delayed. Specifically, IL-1β peaked at 12 h and then gradually decreased, whereas IL-6 and TNF-α exhibited an upward trend at 4 h and remained significantly different from the control group at 24 h. The Western blot results indicated that the protein expression of ICAM-1 and MyD88 showed a delayed increase compared with their mRNA levels. As reported in the literature, the upregulation of protein levels requires time for translation, post-translational processing, and extracellular secretion, which results in a delay relative to mRNA changes[25, 26]. In addition, transcriptomic changes represent immediate intracellular effects, whereas cytokine protein levels reflect the hierarchical progression of the inflammatory cascade and regulation by multiple factors[27, 28].The duration of elevated protein levels may depend on a series of factors such as protein degradation kinetics[29]. Furthermore, different pathogens may induce distinct patterns of inflammatory cytokine elevation and decline in the host[30, 31].

Importantly, this immune stimulation was self-limiting, as the key feature of the “external stimuli alarm” response. Even though we had substantial transcriptional changes on more than 6,000 genes at early points, most of the genes returned to their normal state within 24 h. We did not detect any increase in plasma ALT or AST activity, the gold standard biomarker of hepatocyte damage[32]. Importantly, inflammatory mediators such as IL-1β were induced earlier than TNF-α and IL-6, supporting that IL-1β modulates the expression of TNF-α and IL-6 [33, 34]. The early wave of immune gene expression was robust but tightly controlled in both magnitude and duration, reflecting a highly controlled inflammatory process. Combining the changes in the transcriptome, the inflammatory reaction in the body is relatively short. HE staining of major organs showed no obvious inflammatory lesions. This distinguishes PVC from many delivery vectors that trigger persistently damaging immune responses[35]. The self-resolving nature of this inflammatory response is further supported by the rapid decline of immune-related DEGs after 4 h. Only 753 DEGs remain after 24 h compared to over 3800 DEGs after 2 h. Clustering DEGs into four distinct time-spatial patterns further illustrates how tightly controlled this response is. Most immune-related genes are clear after 24 h. In the extended data, we re-sequenced to assess gene expression changes at 0 h and 72 h. Comparison between the control group and the 72 h sequencing group identified a total of 231 DEGs, including 81 upregulated and 150 downregulated genes.GO enrichment analysis showed that these DEGs were mainly enriched in mitotic cell cycle process, extracellular space, and cell division. KEGG enrichment analysis revealed significant enrichment in pathways including cell cycle, motor proteins, and viral carcinogenesis, with no obvious association with metabolism or inflammation. This precise control of the expression of inflammatory gene expression is a crucial safety feature that prevents the transition from protective immune stimulation to pathological inflammation.

The second step (12-24 h after exposure) showed coordinated downregulation of metabolic homeostasis genes, which reflects physiological adaptation to the previous immune stimulation rather than direct toxicity[36, 37]. We observed the genes of detoxification enzyme families (*Cyp2a4*, *Cyp3a25*, *Cyp2c50* and *Cyp2c54*), glutathione S-transferases (*Gstm1*, *Gsta3* and *Gstm6*), and carboxylesterases (*Ces1f* and *Ces1g*) downregulation after infected PVC 12 h compared with 0 h. In 24 h period, certain genes exhibit sustained upregulation. Among these, some contribute to alleviating inflammatory damage (Orm1, and Orm2) and participating in tissue repair (Mt2), and their recovery or maximal expression may require a longer time frame. Additionally, in the Time-Course Gene Set Analysis, KEGG enrichment found that the PVC infection process also affects other metabolic pathways such as one-carbon metabolism, the interconversion between pentose, and fatty acid elongation pathways. The metabolic gene expression was gradually recovered by 24 h (Fig. 3F, and 5G), forming a distinct “U-shaped” curve in our STEM trend analysis (Profile 1), which strongly supports the interpretation of metabolic function as transient adaptation rather than cytotoxic effect. The synchronous recovery of metabolic pathways, including one-carbon metabolism (*Mthfd1* and *Shmt1/2*), peroxisome function (*Abcd2/3* and *Pex7*), and fatty acid elongation (*Elovl3* and *Acaa2*), indicates a coordinated return to homeostasis rather than a stochastic recovery. All this strongly supports the interpretation of metabolic function as transient adaptation rather than cytotoxic effect. The synchronous recovery of metabolic pathways[38, 39].

Time-course gene set analysis provides insight into the self-limiting response. Profile 42, a bell-shaped “increase-decrease” gene set, contains NF-κB genes (*Myd88*, *Nfkbia* and *Map3k14*), TNF signaling (*Tnfrsf1a* and *Traf1*), and apoptosis (*Bcl2l1* and *Apaf1*).Previous studies have reported that hosts use apoptosis as a defense mechanism to limit infection and eliminate pathogens[40]. Further studies will be required to obtain more experimental data to elucidate the mechanism underlying apoptosis induced by PVC. The downregulation of these pathways by 12–24 h showed the built-in termination mechanisms to prevent long-term immune responses. The U-shaped expression pattern of metabolic genes in Profile 1 shows a transient downregulation followed by a gradual recovery. However, complete recovery could take longer.

Some limitations should be considered. The single-dose design prevents the analysis of dose-response relationships or cumulative effects from repeated administration. While informative, bulk RNA-seq masks cell-type-specific responses. Future single-cell RNA sequencing studies should identify the specific targets of PVC and determine whether different cell types (hepatocytes, Kupffer cells, and endothelial cells) contribute differently to the observed self-limiting response. Although most gene responses typically resolve within 24 h, a small subset of genes remains incompletely recovered. Therefore, long-term studies must be carried out to rule out immune priming or delayed responses. Moreover, the mechanism of precise timing of PVC-induced transcription and, notably, the factors that ensure complete resolution of immune and metabolic changes need further studies.

Single-dose exposure to PVC induces a self-limiting acute hepatic response that reprograms immune and metabolic pathways biphasically. The innate immune response typically recovers within 24 h, while the complete recovery of metabolism-related genes may take a longer time. While the present findings demonstrate an absence of overt acute hepatotoxicity and only transient transcriptional changes following PVC administration, these data are limited to a single dose and a relatively short observation period. Accordingly, our results support that PVC exhibits favorable acute liver safety profiles at the tested dose, particularly for applications requiring transient activity and limited exposure.

## Supplemental Materials

Supplementary data for this paper are available on-line only at http://jmb.or.kr.



## Figures and Tables

**Table 1:** The primer sequences chosen in the qRT-PCR.

Gene Name	Forward Primer (5'-3')	Reverse Primer (5'-3')
*ILIRI*	GCCAAGGTGGAGGACTCAGG	CACAAGCCAGGGTCATTCTCTAAC
*IMTHFD1*	CACGCCATCACTGCTGCTAAC	GTACCAGACGATTAAAGAGAGCCTTG
*GSIM1*	CCCTGACTTTGTGGCTGTTCC	TCTGGCTTCTGCTTCTCCTGAG
*CYP3A25*	ACTCCTGTCTCCAACCTTCACC	TCTTCTTCTCGTCTCAAGTTTCTCAC
*MYD88*	CGGCAACTAGAACAGACAGACTATC	TCTCAATTAGCTCGCTGGCAATG
*ICAM1*	TGCCTCTGAAGCTCGGATATACC	CGAACTCCTCAGTCACCTCTACC
*GAPDH*	GGTTGTCTCCTGCGACTTCA	TGGTCCAGGGTTTCTTACTCC

**Table 2:** Top 20 up-/downregulated genes from each time point comparison for mouse liver tissue.

0 h VS 2 h				0 h VS 4 h				0 h VS 12 h				0 h VS 24 h			
gene Log_2_FC	Padjust	Regulated	gene	Log_2_FC	Padjust	Regulated	gene	Log_2_FC	Padjust	Regulated	gene	Log_2_FC	Padjust	Regulated
*Steap4*	6.647	<0.00001	up	*Steap4*	6.345	<0.00001	up	*Cdkn1a*	4.632	1.25E-161	up	*Orm1*	3.350	5.39E-99	up
*Pfkfb3*	5.729	<0.00001	up	*Igfbp1*	4.503	<0.00001	up	*Cxcl9*	4.363	2.43E-136	up	*Hp*	3.476	1.83E-80	up
*Il1rn*	7.541	<0.00001	up	*Cdkn1a*	6.428	<0.00001	up	*Cyp2a5*	-5.472	<0.00001	down	*Orm2*	5.703	1.83E-79	up
*Il1r1*	4.753	2.29E-303	up	*Il1r1*	4.957	<0.00001	up	*Gsta3*	-4.528	<0.00001	down	*Hpx*	3.247	1.36E-77	up
*Cpne8*	5.525	3.37E-293	up	*Tifa*	5.853	1.40E-265	up	*Car3*	-6.194	<0.00001	down	*Itgam*	4.734	2.86E-62	up
*Cdkn1a*	5.516	8.69E-273	up	*Nopchap1*	3.002	1.37E-206	up	*Cyp2c50*	-3.864	<0.00001	down	*Isyna1*	3.584	5.60E-49	up
*Tifa*	5.588	2.92E-270	up	*Eif4ebp3*	5.669	5.36E-200	up	*Serpina6*	-5.491	<0.00001	down	*Saa3*	11.184	5.09E-44	up
*Tlr2*	6.233	1.86E-260	up	*Cpne8*	5.104	9.84E-189	up	*Hamp2*	-7.165	2.58E-300	down	*Apcs*	2.006	1.24E-37	up
*Igfbp1*	4.820	3.57E-260	up	*Serpina3n*	4.858	2.31E-188	up	*Inmt*	-5.684	2.21E-274	down	*Mt2*	6.356	1.47E-31	up
*Cd14*	6.619	3.25E-235	up	*Ets2*	3.379	2.14E-187	up	*Cyp2c54*	-4.185	1.50E-260	down	*Itih3*	2.289	1.24E-28	up
*Snx10*	4.104	9.37E-234	up	*Gbp2*	4.241	1.94E-180	up	*Acaa1b*	-4.590	2.61E-250	down	*Per1*	3.215	8.24E-28	up
*Susd6*	3.514	1.96E-231	up	*Icam1*	4.117	4.74E-172	up	*Ces1g*	-3.690	1.67E-234	down	*Serpina12*	3.386	5.56E-27	up
*Plscr1*	4.536	2.24E-216	up	*Serpina3c*	6.636	7.91E-170	up	*Cyp3a25*	-4.416	2.08E-212	down	*Hectd2os*	-2.458	2.48E-59	down
*Nopchap1*	3.478	4.23E-208	up	*Fndc3b*	3.397	1.44E-165	up	*Gstm6*	-3.479	5.18E-198	down	*Crybb3*	4.664	4.16E-57	down
*Sgms1*	3.617	3.71E-189	up	*Gbp2b*	5.131	2.83E-164	up	*Upp2*	-5.069	2.32E-192	down	*Aqp8*	-1.934	2.36E-50	down
*Myd88*	3.489	7.28E-172	up	*Cxcl9*	6.147	4.42E-160	up	*Gstm1*	-3.624	7.26E-183	down	*Prom1*	-2.514	4.43E-34	down
*Pik3ap1*	3.327	2.86E-171	up	*Manf*	2.640	1.41E-151	up	*Ces1f*	-5.759	1.88E-175	down	*Gsta2*	-3.822	3.11E-33	down
*Tiparp*	4.229	2.24E-170	up	*Coq8a*	-4.86	0.00E+00	down	*Ugt2b1*	-2.909	1.97E-172	down	*Dusp1*	-2.008	8.39E-30	down
*Selp*	6.136	6.10E-162	up	*Aqp8*	-4.14	1.09E-195	down	*Cyp2a4*	-4.228	8.13E-162	down	*Ehhadh*	-1.669	5.85E-28	down
*Icam1*	4.380	2.26*-160	up	*Ddc*	-3.86	4.97E-170	down	*Afmid*	-4.537	3.48E-140	down	*Ces2c*	-2.511	1.05E-27	down

## References

[1] Wang X, Cheng J, Shen J, Liu L, Li N, Gao N (2022). Characterization of photorhabdus virulence cassette as a causative agent in the emerging pathogen *Photorhabdus asymbiotica*. Science China. Life Sci..

[2] Jiang F, Li N, Wang X, Cheng J, Huang Y, Yang Y (2019). Cryo-EM Structure and assembly of an extracellular contractile injection system. Cell.

[3] Xu J, Ericson CF, Lien Y-W, Rutaganira FUN, Eisenstein F, Feldmüller M, King N (2022). Identification and structure of an extracellular contractile injection system from the marine bacterium *Algoriphagus machipongonensis*. Nat. Microbiol..

[4] Vladimirov M, Gautam V, Davidson AR (2022). Identification of the tail assembly chaperone genes of T4-Like phages suggests a mechanism other than translational frameshifting for biogenesis of their encoded proteins. Virology.

[5] Sarris PF, Ladoukakis ED, Panopoulos NJ, Scoulica EV (2014). A phage tail-derived element with wide distribution among both prokaryotic domains: a comparative genomic and phylogenetic study. Genome Biol. Evol..

[6] Yang G, Dowling AJ, Gerike U, ffrench-Constant RH, Waterfield NR (2006). Photorhabdus virulence cassettes confer injectable insecticidal activity against the wax moth. J. Bacteriol..

[7] Kreitz J, Friedrich MJ, Guru A, Lash B, Saito M, Macrae RK (2023). Programmable protein delivery with a bacterial contractile injection system. Nature.

[8] Kreitz J, Yang V, Friedrich MJ, Pham J, Macrae RK, Zhang F. 2025. Targeted delivery of diverse biomolecules with engineered bacterial nanosyringes. *Nat. Biotechnol.* https://doi.org/10.1038/s41587-025-02774-x. 10.1038/s41587-025-02774-x 40796978 PMC13368577

[9] Zheng Z, Lu Y, Wu H, Lam PU, Sun X, Song Y (2024). Clinical outcomes of Omicron infection and vaccine acceptance among pediatric liver transplant recipients: Insights from a cross-sectional survey. Virol. J..

[10] Kubes P, Jenne C (2018). Immune Responses in the Liver. Ann. Rev. Immunol..

[11] Wang Q, Zhang Y, Zhu A (2025). Drug Metabolism and Toxicological Mechanisms. Toxics.

[12] Li L, Cui L, Lin P, Liu Z, Bao S, Ma X (2023). Kupffer-cell-derived IL-6 is repurposed for hepatocyte dedifferentiation via activating progenitor genes from injury-specific enhancers. Cell Stem Cell.

[13] Li B, Dewey CN (2011). RSEM: Accurate transcript quantification from RNA-Seq data with or without a reference genome. BMC Bioinformatics.

[14] Shan L, Chen Y, An G, Tao X, Qiao C, Chen M (2024). Polyphyllin I exerts anti-hepatocellular carcinoma activity by targeting ZBTB16 to activate the PPARγ/RXRα signaling pathway. Chinese Med..

[15] Liu T, Zhang L, Joo D, Sun S-C (2017). NF-κB signaling in inflammation. Signal Transduct. Target. Ther..

[16] Sahu U, Biswas D, Prajapati VK, Singh AK, Samant M, Khare P (2021). Interleukin-17-A multifaceted cytokine in viral infections. J. Cell. Physiol..

[17] Icard P, Lincet H, Wu Z, Coquerel A, Forgez P, Alifano M, Fournel L (2021). The key role of Warburg effect in SARS-CoV-2 replication and associated inflammatory response. Biochimie.

[18] Chermahini FA, Arvejeh PM, Marincola FM, Ahmad S, Naderian R, Pajand O, *et al*. (2025). Investigating how dengue virus-induced metabolic changes affect the host immune response and how to develop Immunomodulatory strategies. *Virol. J.* **22:** 117. https://doi.org/10.1186/s12985-025-02745-3. 10.1186/s12985-025-02745-3 40281578 PMC12023479

[19] Yokota S-I, Okabayashi T, Fujii N (2010). The battle between virus and host: Modulation of Toll-like receptor signaling pathways by virus infection. Mediators Inflamm..

[20] Chen R, Zou J, Chen J, Zhong X, Kang R, Tang D (2025). Pattern recognition receptors: function, regulation and therapeutic potential. Signal Transduct. Target. Ther..

[21] Tahtinen S, Tong A-J, Himmels P, Oh J, Paler-Martinez A, Kim L (2022). IL-1 and IL-1ra are key regulators of the inflammatory response to RNA vaccines. Nat. Immunol..

[22] Kombe Kombe AJ, Fotoohabadi L, Gerasimova Y, Nanduri R, Lama Tamang P, Kandala M (2024). The role of inflammation in the pathogenesis of viral respiratory infections. Microorganisms.

[23] Huang M, Xu M, Han J, Ke E, Niu X, Zhang Y (2025). Enhancing MyD88 oligomerization is one important mechanism by which IBDV VP2 induces inflammatory response. PLoS Pathog..

[24] Viñán Garcés AE, Cáceres E, Gómez JO, Martín-Loeches I, Reyes LF (2024). Inflammatory response to SARS-CoV 2 and other respiratory viruses. Expert Rev. Anti-Infective Ther..

[25] Brockmann R, Beyer A, Heinisch JJ, Wilhelm T (2007). Posttranscriptional expression regulation: what determines translation rates?. PLoS Comput. Biol..

[26] Cheng Z, Teo G, Krueger S, Rock TM, Koh HWL, Choi H (2016). Differential dynamics of the mammalian mRNA and protein expression response to misfolding stress. Mol. Syst. Biol..

[27] Salerno F, Paolini NA, Stark R, von Lindern M, Wolkers MC (2017). Distinct PKC-mediated posttranscriptional events set cytokine production kinetics in CD8^+^ T cells. Proc. Natl. Acad. Sci. USA.

[28] Guillemin A, Kumar A, Wencker M, Ricci EP (2021). Shaping the Innate Immune Response Through Post-Transcriptional Regulation of Gene Expression Mediated by RNA-Binding Proteins. Front. Immunol..

[29] Rioja I, Bush KA, Buckton JB, Dickson MC, Life PF (2004). Joint cytokine quantification in two rodent arthritis models: Kinetics of expression, correlation of mRNA and protein levels and response to prednisolone treatment. Clin. Exper. Immunol..

[30] Chen L, Li L, Huang C, Cao X, Jiang Y (2024). Dynamic mRNA network profiles in macrophages challenged with lipopolysaccharide. Am. J. Transl. Res..

[31] Hu W-C (2020). A Framework of all discovered immunological pathways and their roles for four specific types of pathogens and hypersensitivities. Front. Immunol..

[32] Nie W, Xu F, Zhou K, Yang X, Zhou H, Xu B (2022). Stearic acid prevent alcohol-induced liver damage by regulating the gut microbiota. Food Res. Int. (Ottawa, Ont.).

[33] Pyrillou K, Burzynski LC, Clarke MCH (2020). Alternative Pathways of IL-1 Activation, and Its Role in Health and Disease. Front. Immunol..

[34] Semino C, Carta S, Gattorno M, Sitia R, Rubartelli A (2018). Progressive waves of IL-1β release by primary human monocytes via sequential activation of vesicular and gasdermin D-mediated secretory pathways. Cell Death Dis..

[35] Chen Q, Yang Z, Liu H, Man J, Oladejo AO, Ibrahim S (2024). Novel drug delivery systems: an important direction for drug innovation research and development. Pharmaceutics.

[36] Xie X, Liu P-S, Percipalle P (2019). Analysis of global transcriptome change in mouse embryonic fibroblasts after dsDNA and dsRNA viral mimic stimulation. Front. Immunol..

[37] Hu T, Liu C-H, Lei M, Zeng Q, Li L, Tang H (2024). Metabolic regulation of the immune system in health and diseases: Mechanisms and interventions. Signal Transduct. Target. Ther..

[38] Xia W, Mao Y, Xia Z, Cheng J, Jiang P (2025). Metabolic remodelling produces fumarate via the aspartate-argininosuccinate shunt in macrophages as an antiviral defence. Nat. Microbiol..

[39] Palmer CS (2022). Innate metabolic responses against viral infections. Nat. Metab..

[40] Zhang Z, Xiao K, Wang S, Ansari AR, Niu X, Yang W (2022). Visfatin is a multifaceted molecule that exerts regulation effects on inflammation and apoptosis in RAW264.7 cells and mice immune organs. Front. Immunol..

