# Supplementary material for: Engineered Bacterial Nanosyringes Induce Transient and Controllable Hepatic Immune-Metabolic Responses
Source: J Microbiol Biotechnol. 2026 May 25;36:e2601002. doi: 10.4014/jmb.2601.01002 (PMC13222765; doi:10.4014/jmb.2601.01002)
Supplement: Supplementary file 1 [file jmb-36-e2601002-supple.pdf]

Supplementary Table S1. RNA Sequencing Data Analysis: Quality Control, Assembly, and Mapping.

Sample	Total reads	Total mapped	Multiple mapped	Unique mapped
0 h a	39290004	37976202(96.66%)	3079418(7.84%)	34896784(88.82%)
0 h b	43020652	41787909(97.13%)	3751827(8.72%)	38036082(88.41%)
0 h c	48883954	47428096(97.02%)	4294501(8.79%)	43133595(88.24%)
2 h a	44188802	42690249(96.61%)	3464668(7.84%)	39225581(88.77%)
2 h b	43033840	41635283(96.75%)	3455823(8.03%)	38179460(88.72%)
2 h c	42861522	41595328(97.05%)	3245355(7.57%)	38349973(89.47%)
4 h a	43711488	42288859(96.75%)	3485015(7.97%)	38803844(88.77%)
4 h b	43942210	42449942(96.6%)	3536799(8.05%)	38913143(88.56%)
4 h c	45568116	44112346(96.81%)	3540726(7.77%)	40571620(89.04%)
12 h a	42965240	41681402(97.01%)	3210744(7.47%)	38470658(89.54%)
12 h b	43329960	42090195(97.14%)	3261871(7.53%)	38828324(89.61%)
12 h c	43102930	41689529(96.72%)	2986282(6.93%)	38703247(89.79%)
24 h a	39901098	38775972(97.18%)	2791841(7.0%)	35984131(90.18%)
24 h b	43228216	42096213(97.38%)	3204437(7.41%)	38891776(89.97%)
24 h c	45119798	43890228(97.27%)	3269900(7.25%)	40620328(90.03%)

Supplementary Table S2 Statistical Analysis of Changes in Key Genes Related to Innate Immunity and Metabolism at Different Time Points

	baseline VS 2hours			baseline VS 4hoursh			baseline VS12hours			baseline VS24hours		
Gene name	Log_2FC	Padjust	Significant	Log_2FC	Padjust	Significant	Log_2FC	Padjust	Significant	Log2FC	Padjust	Significant
Steap4	6.647	<0.00001	yes	6.345	<0.00001	yes	3.184	1.87E-58	yes	1.525	6.06E-06	yes
Il1rn	7.541	<0.00001	yes	6.944	1.45E-143	yes	4.621	1.33E-84	yes	2.458	1.10E-06	yes
Il1r1	4.753	2.29E-303	yes	4.957	0.00E+00	yes	2.196	9.67E-21	yes	1.555	0.045	no
Tifa	5.588	2.92E-270	yes	5.588	2.922E-270	yes	3.128	2.82E-32	yes	1.269	1.45E-06	yes
Tlr2	6.233	1.86E-260	yes	4.256	2.12E-118	yes	1.373	5.29E-10	yes	0.980	5.929E-06	no
Cd14	6.619	3.25E-235	yes	6.642	1.31E-114	yes	5.243	3.47E-20	yes	2.195	1.31E-11	yes
Plscr1	4.536	2.24E-216	yes	3.384	5.87E-78	yes	0.530	0.005	no	-0.323	0.306	no
Myd88	3.489	7.28E-172	yes	2.852	4.46E-101	yes	0.786	1.869E-06	no	0.291	0.233	no
Tiparp	4.229	2.24E-170	yes	3.709	2.59E-93	yes	-0.034	0.926	no	-0.694	0.027	no
Selp	6.136	6.10E-162	yes	4.519	3.18E-47	yes	2.000	4.50E-07	yes	0.503	0.457	no
Icam1	4.380	2.26E-160	yes	4.117	4.74E-172	yes	1.874	4.38E-31	yes	1.285	5.969E-11	no
Cdkn1a	5.516	8.69E-273	yes	6.428	<0.00001	yes	4.632	1.25E-161	yes	2.700	1.20E-23	yes
Serpina3n	5.668	1.38E-138	yes	4.858	2.31E-188	yes	3.887	2.24E-107	yes	2.003	0.145	no
Gbp2b	4.325	3.80E-126	yes	5.131	2.83E-164	yes	-0.017	0.977	no	-0.390	0.389	no
Cyp2a5	-0.650	0.002	no	-1.736	5.949E-24	no	-5.472	<0.00001	yes	-0.710	0.382	no
Gsta3	-0.543	0.008	no	-1.087	5.012E-21	no	-4.528	<0.00001	yes	-1.859	1.910E-11	no
Cyp2c50	-0.158	0.445	no	-1.030	2.803E-09	no	-3.864	<0.00001	yes	-2.048	2.61E-17	yes
Cyp2c54	-0.185	0.100	no	-0.945	2.954E-05	no	-4.185	1.50E-260	yes	-1.878	2.964E-10	no
Ces1g	0.038	0.885	no	-1.037	4.711E-13	no	-3.690	1.67E-234	yes	-1.717	1.813E-07	no
Cyp3a25	-0.235	0.256	no	-1.187	3.907E-17	no	-4.416	2.08E-212	yes	-1.082	7.019E-10	no
Gstm6	-0.236	0.147	no	-0.529	3.538E-04	no	-3.479	5.18E-198	yes	-1.988	1.027E-36	no
Gstm1	-0.104	0.683	no	-0.664	1.590E-07	no	-3.624	7.26E-183	yes	-2.243	7.49E-24	yes

Supplementary Table S2 Statistical Analysis of Changes in Key Genes Related to Innate Immunity and Metabolism at Different Time Points

	baseline VS 2hours			baseline VS 4hoursh			baseline VS12hours			baseline VS24hours		
Gene name	Log_2FC	Padjust	Significant	Log_2FC	Padjust	Significant	Log_2FC	Padjust	Significant	Log2FC	Padjust	Significant
Ces1f	-0.373	0.139	no	-1.117	7.672E-08	no	-5.759	1.88E-175	yes	-2.299	1.01E-24	yes
Cyp2a4	-0.125	0.506	no	-0.917	7.774E-18	no	-4.228	8.13E-162	yes	-1.400	0.225	no
Orm1	1.893	5.426E-33	no	2.660	1.24E-68	yes	3.225	5.70E-104	yes	3.350	5.39E-99	yes
Orm2	3.122	2.476E-09	yes	3.237	1.18E-30	yes	4.697	3.47E-23	yes	5.703	1.83E-79	yes
Isyna1	1.047	1.232E-02	no	1.136	6.78E-05	yes	3.090	1.24E-52	yes	3.584	5.60E-49	yes
Mt2	8.281	2.977E-69	yes	8.557	5.16E-75	yes	6.597	3.37E-41	yes	6.356	1.47E-31	yes
